# Study of Zn‐Astrakanite/CuO/ZnO Nanocomposite Using *Tribulus terrestris* Aqueous Extract, and Their Structural, Optical, Morphological, Dielectric, and Bacterial Properties

**DOI:** 10.1002/jemt.70041

**Published:** 2025-07-21

**Authors:** M. Kalaiyarasi, M. Mani, R. Harikrishnan, N. Bharathiraja, J. Kishorkumar, L. Sibali, K. Kaviyarasu

**Affiliations:** ^1^ PG and Research Department of Physics Arignar Anna Govt. Arts College Cheyyar India; ^2^ PG and Research Department of Physics Kalaignar Karunanidhi Govt. Arts College Tiruvannamalai India; ^3^ Department of Physics St. Joseph College of Engineering Sriperumbudur India; ^4^ PG and Research Department of Zoology Muthurangam Government Arts College (Autonomous) Vellore Tamil Nadu India; ^5^ Department of Environmental Sciences College of Agriculture and Environmental Sciences, University of South Africa Florida South Africa; ^6^ UNESCO‐UNISA Africa Chair in Nanosciences/Nanotechnology Laboratories, College of Graduate Studies, University of South Africa (UNISA) Pretoria South Africa

**Keywords:** antibacterial activity, biomass, biosynthesis composites, electron microscopy, Zn astrakanite structure

## Abstract

Zn‐astrakanite/CuO/ZnO nanocomposite was prepared using environmentally friendly and biogenically derived approaches through nanoengineering. *Tribulus terrestris* plant extract was used in this study to synthesize the Zn‐astrakanite/CuO/ZnO nanocomposite. For the first time, the formation of a Zn‐substituted astrakanite structure was achieved using a green synthesis method guided by the reducing, capping, and chelating agents present in the *Tribulus terrestris* plant extract. By using Ultraviolet visible (UV–vis) spectroscopy, we observe a characteristic peak at 376 nm that indicates the presence of ZnO nanoparticles, and in addition, the appearance of green color confirmed the presence of CuO nanoparticles in colloidal solutions. An analysis of Rietveld refined powder X‐ray diffraction (PXRD) provides insight into the novel crystalline structure of Zn‐astrakanite. It was found from the Rietveld refinement results that the synthesized nanocomposite composed of CuO, Zn‐astrakanite, and ZnO crystalline phases with compositions 56.26%, 31.49%, and 12.26%, respectively. The green color appearance of the synthesized nanocomposite in colloidal solution was due to the presence of CuO nanoparticles as a major composition and surface plasmon resonance (SPR) characteristic. As revealed by scanning electron microscopy (SEM) analysis, nanoparticles have blade‐like morphologies due to ZnO and some randomly shaped crystallites. Fourier transform infrared spectroscopy (FTIR) analysis provides functional information about the synthesized compound, and we detected CuO and ZnO nanocomposites by observing the metal oxide fingerprint regions at 523 cm^−1^ and 472 cm^−1^. The synthesized nanocomposites were reported to have good bactericidal activity and electrical conductivity when tested for antibacterial activity and dielectric behavior, respectively, which were discussed in detail.

## Graphical abstract

Upon UV–vis characterization of biosynthesized Cu doped ZnO nanoparticles, it was found that their bandgap energy was 2.73 eV. In the biosynthesized nanoparticles, metal oxides and functional groups were identified by FTIR analysis. In both Zn‐O and Cu‐O lattices, 472 cm^−1^ and 523 cm^−1^ were observed, respectively. According to PXRD analysis, Zn astrakanite, CuO (Tenorite), and ZnO (Zincite) are new structures. The nanostructure resembled a blade when viewed through SEM. A final step involved finding that *S. aureus* and *E. coli* had antibacterial properties. It appears that the synthesized compound has good antibacterial properties at different concentrations, including 50 μg, 100 μg, 250 μg, and 500 μg. Based on these results, the green synthesized Cu doped ZnO nanoparticles have been tested in a variety of methods.

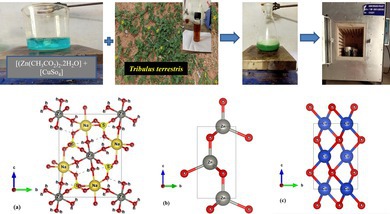

**Summary**

- We present a novel method for refining disodium zinc bis(sulfate) tetrahydrate at low temperature. Zn‐astrakanite is a new crystalline form and it's an upgrade of data previously reported.
- PXRD analysis revealed a new structure called Zn astrakanite, CuO (Tenorite), and ZnO (Zincite).
- Narrow band of (O—C=O) Cu—O and Zn—O lattice can be observed in the metal oxide region at 523 and 472 cm^−1^.
- The concentration which led to clear inhibition was above all concentrations, which suggests a good level of bacterial resistance.

## 1 Introduction

The field of nanotechnology is one of the most effective research subjects available today. Worldwide, researchers use biological approaches for synthesizing metal oxide nanoparticles, which are eco‐friendly and sustainable (Nasrollahzadeh et al. 2019). There was an extensive amount of high purity ZnO nanorods obtained in a study by Fang et al. Interestingly, the as‐synthesized ZnO nanorods had diameters ranging from 40 to 400 nm, allowing them to form 3D networks for sensing applications (Fang et al. 2009; Murmu et al. 2015). Interestingly, Murmu et al.'s work describes a red shift in defect emission at low temperatures, making the green emissions appear orange and yellow, indicating suppression of the deep level (Murmu et al. 2011). Besides being the 23rd element to be found naturally in the Earth's crust, it is also one of the most used materials on the planet. In addition to being the second most prevalent metal in the human body, it plays a significant role in biological processes. In terms of readily available zinc compounds, only zinc oxide is recognized by the US Food and Drug Administration as generally safe (GRAS). A large bandgap (~3.3 eV) and considerable excitation binding energy make zinc oxide nanoparticles good *n*‐type semiconductors (Zhang 2005; Osredkar and Sustar 2011; He et al. 2019; Caglar et al. 2009; Król et al. 2017). Moreover, copper is one of the earth's most abundant elements (Crisan et al. 2022). Due to its many properties, including superior electrical and thermal conductivity, excellent corrosion resistance, and improved malleability, the material is ideally suited for carrying out current research (Fan et al. 2022; Murthy et al. 2020). It has been found that copper nanoparticles are suitable for a variety of applications, such as solar cells, gas sensors, light emitting diodes, electronics, emitters, cathode materials, and other uses (Lin et al. 2023; Steinhauer 2021). There are several factors that influence copper‐based nanoparticles, including high oxidative damage, cell (DNA) damage, cytotoxicity, and cytotoxicity (Karlsson et al. 2008; Strauch et al. 2017). A transition metal (Cu, Mn, Cr and Fe) and biomolecule can be added to zinc oxide nanoparticle surfaces to improve efficiency and functionality (Khan et al. 2018). The fabrication of ZnO nanoparticles has been accomplished using a variety of materials, but copper has proven to be the best material to dope with zinc. Because of its π‐electron structure, its valence bond to ZnO can easily overlap. Copper oxide and zinc oxide nanocomposite or doping can enhance their physical and chemical properties, resulting in a larger surface area and smaller particle size, thereby increasing their antioxidant and bactericidal effects (Bhosale et al. 2022; Hojaghani et al. 2014). Sonochemical synthesis, sol–gel synthesis, microwave irradiation, thermal decomposition, chemical reduction, and others have been found to produce nanoparticles with good shape and size control. Environmentally hazardous byproducts are produced by these methods (Tian et al. 2012; Anastas 1999; Sadeghi and Gholamhoseinpoor 2015). However, compared to chemical methods, organic methods were considered eco‐friendly and there were no hazardous by‐products. Upon reduction of metal ions into elemental ions, the plant extract acts as a stabilizing agent and a reducing agent (Singh et al. 2018; Brown 2017). In this study, we use plant extracts of *Tribulus terrestris* . This plant part is commonly used as a medicine to the treatment of gallstones and kidney stones. *Tribulus terrestris* decoction (boiled extract) is often used to improve kidney function. We can remove liver stones naturally without experiencing any side effects from it (Alok et al. 2013; Akbar et al. 2017). In addition to purifying our blood cells, it also cleanses our intestinal tract.

Additionally, biological techniques use biomass‐derived bioactive compounds such as polyphenols, flavonoids, and proteins for the formation of bulk structured particles to nanoscale level distribution of particles. These compounds are naturally derived from plants and are available in many parts such as leaves, roots, and flowers. The biological compounds that are responsible for converting bulk structured particles into nanostructured particles are known as reducing and stabilizing agents. The chemical compounds that constitute excess electrons act as donors, which are known as reducing agents. During the synthesis process, they interact with the metal ions by donating electrons to the metal ions, resulting in the conversion of the higher oxidation state of the metal ion to a lower state. During this process, metal oxide nanoparticles are formed. After the formation of metal oxides (*reduction process*), high surface energy may create aggregation, resulting in the distortion of the formed metal oxide structure at the nanoscale level. Therefore, to avoid such difficulties, capping agents are much needed to stabilize the formed metal oxide nanoparticles. They stabilize them after binding with the surface region of metal oxide nanoparticles, which act as a protective layer. They are also one of the key components responsible for deciding the size and shape of nanoparticles. The compounds that are naturally present in the plant part biomaterials such as leaf, root, and flower (Semerdjieva and Zheljazkov 2019). The reducing and capping agents present in *Tribulus terrestris* leaf extracts, responsible for the synthesis of ZnO and CuO nanoparticles, typically include various phytochemicals such as: Reducing agents: (i) Flavonoids, (ii) Phenolic compounds, (iii) Alkaloids, (iv) Ascorbic acid. Antioxidants present in flavonoids act as natural reducing agents, capable of converting bulk ZnO or CuO particles into nanoparticles. Phenolic compounds and ascorbic acid donate electrons to metal ions, causing their reduction. The capping agents present in the leaf extracts, such as tannins, saponins, and proteins, form a protective layer around the ZnO and CuO nanoparticles, thereby increasing their stability (Hammoda et al. 2013).

Table 1 reveals that CuO/ZnO nanocomposites can be synthesized using various plant leaf extracts, and their potential for environmental applications is summarized. The extracts of some plants, such as *Zingiber officinale* and Costus igneus, act as toxic agents against microbial species, while plants like *Artemisia vulgaris* and *Croton macrostachyus* are frequently used as antioxidant agents, which are beneficial for environmental and medical uses. Several studies have reported the antimicrobial applications of CuO and ZnO nanoparticles. Therefore, synthesizing CuO/ZnO nanocomposites using medicinal plants can enhance these antimicrobial applications. In this study, the addition of the compound NaOH and CuSO_4_ resulted in the formation of an additional structure, Zn substituted astrakanite. Cations Zn^2+^ are incorporated into a structure known as astrakanite, forming Zn‐astrakanite. A typical unit cell structure of astrakanite is composed of sodium, calcium, and sulfate ions. In this work, the green synthesis method successfully facilitated the formation of Zn‐astrakanite. Phytochemicals in plant extracts interact with zinc salts, reducing them to Zn^2+^ ions, which are easily incorporated into the astrakanite structure. The nucleation and growth of Zn‐substituted astrakanite are initiated when Zn^2+^ ions bind with other ions such as SO4^2−^ and ^+^Na. This formation is further guided by the capping and chelating agents present in leaf extracts, which influence the control of crystal size, shape, and growth directions.

**TABLE 1:** A comparison between the ZnO/CuO nanocomposite and various plant extracts used as reducing and capping agents.

Nanocomposite	Name of the plant used	Particle size/morphology	Applications	References
Zn‐astrakanite/CuO/ZnO	*Tribulus terrestris*	Some blade‐like and some random shaped crystalline structure	Antimicrobial and Dielectric	This study
ZnO/CuO	*Achyranthes aspera*	Nanocapsules like morphology for ZnO and Spherical shaped nanoparticles for CuO	Dielectric applications	Pandey and Choubey (2024)
ZnO/CuO	*Artemisia vulgaris*	22.50 nm/	Wastewater treatment degradation of methylene blue	Nepal et al. (2024)
CuO/Ag/ZnO	Ziziphus spina‐christi	Spherical dot like morphology/7.11 ± 0.67 nm	Antimicrobial activities against *Escherichia coli* and *Staphylococcus aureus*	El‐Sawaf et al. (2024)
CuO/ZnO	*Dovyalis caffra*	Without a definite shape (Densely packed)	Antioxidant and anticancer activities	Adeyemi et al. (2022)
ZnO/CuO	*Croton macrostachyus*	Spherical shaped	Wastewater treatment Degradation of methylene blue	Endeshaw et al. (2024)
ZnO/CuO	*Croton Macrostachyus*	Spherical and rod like	Methylene blue degradation and antimicrobial activities	Masho et al. (2024)
CuO/ZnO	V. Sinaiticum Benth	Plate‐like nanostructures	Degradation of Methylene blue and 4‐Nitrophenol	Bekru et al. (2022)
ZnO/CuO	*Tragia involucrata* L	Spherical shaped/39 nm	Photocatalytic degradation of Rhodamine B	Jeevarathinam and Asharani (2024)
ZnO/CuO	*Zingiber officinale* Rhizome	18.41 to 20.50 nm	Antimicrobial studies	Takele et al. (2023)
ZnO/CuO	*Annona glabra*		Antimicrobial and Photocatalytic degradation of organic pollutants	Nguyen et al. (2023)
ZnO/CuO	Costus igneus		Photocatalytic degradation of organic pollutants	Nithiyavathi et al. (2021)
ZnO/CuO	Parsley	Spherical shape/70 nm	Anticoccidial Application	Hamad et al. (2023)

In this study, we aim to study the biological activities of Zn‐astrakanite/CuO/ZnO nanocomposite synthesized by *Tribulus terrestris* aqueous extract Using *Tribulus terrestris* as a biomaterial for Zn‐astrakanite synthesis does not appear to have been reported in the literature. In addition, the synthesized Zn‐astrakanite/CuO/ZnO nanocomposite is characterized by UV–vis, XRD, SEM, and FTIR analysis, and their antimicrobial and dielectric properties are studied.

## 2 Materials and Methods

### 2.1 Materials

During a field trip to Thenvanakkambadi village in Thiruvannamalai District, Tamil Nadu, India, fresh plants of *Tribulus terrestris* were collected. The chemical ingredients used in the preparation of Zn‐astrakanite/CuO/ZnO nanocomposite include copper, sodium hydroxide, and zinc acetate. Analytical grade copper sulfate (CuSO_4_), sodium hydroxide (NaOH), and zinc acetate [Zn(CH_3_CO_2_)_2_] chemicals were purchased without any further purification.

### 2.2 Preparation of *Tribulus terrestris* Aqueous Extract

**FIGURE 1:**
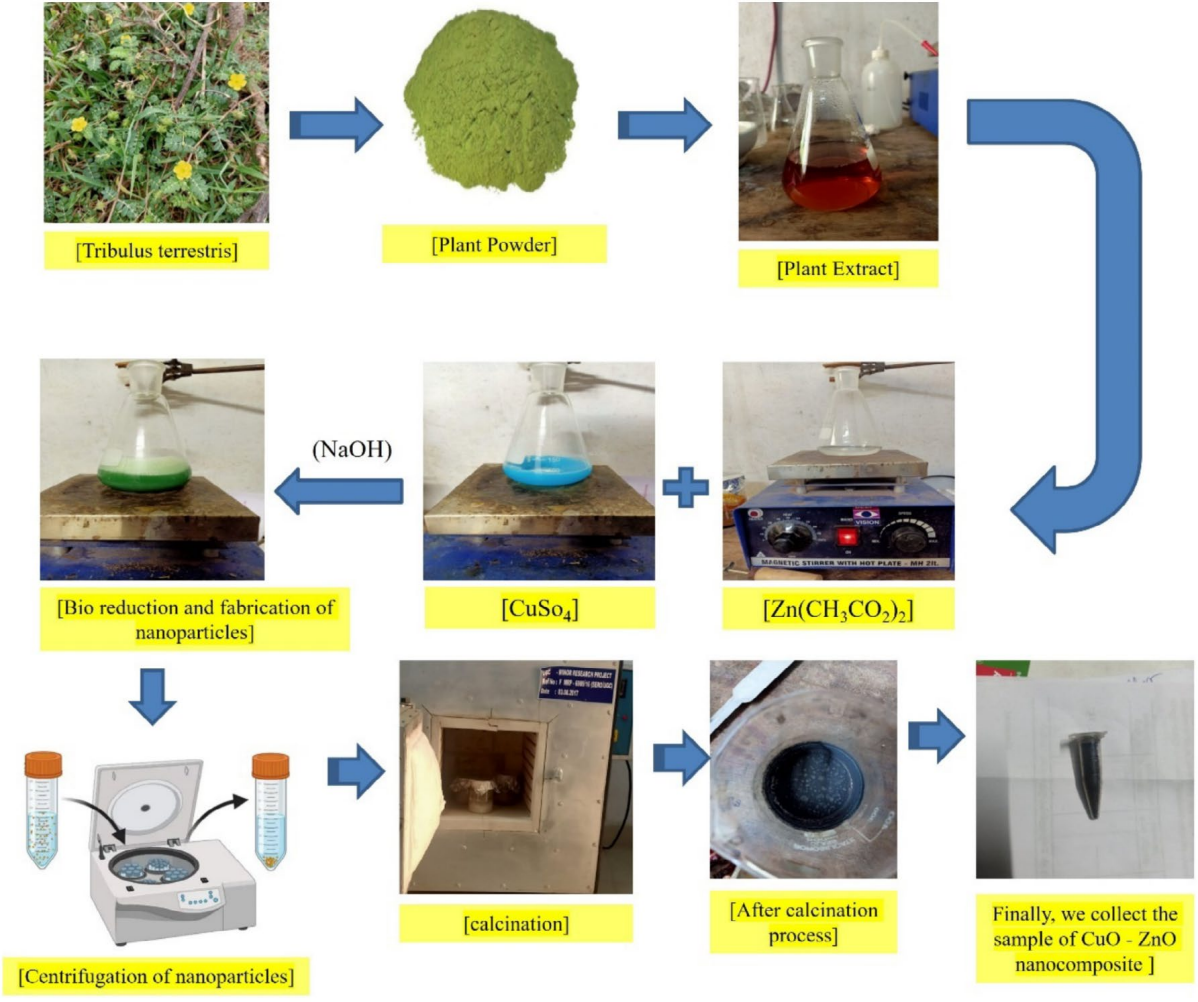
Schematic mechanism for the production of Zn‐astrakanite/CuO/ZnO nanocomposite using *Tribulus terrestris*.

Freshly collected *Tribulus terrestris* were thoroughly cleaned with deionized water several times after collecting. As soon as it had been crushed, it was shade dried and shade dried again. It was mixed with 100 mL of deionized water after adding 10 g of powdered *Tribulus terrestris* . After that, it was boiled for 30 min at 80°C. A Whatman filter paper was used to filter the extracted substance, which then had to be kept in a refrigerator below 4°C for some time after that. As shown in Figure 1, the aqueous extract was prepared.

### 2.3 *Biosynthesis of* Zn‐Astrakanite/CuO/ZnO Nanocomposite

In order to synthesize the Zn‐astrakanite/CuO/ZnO nanocomposite, aqueous extracts of *Tribulus terrestris* were used to co‐precipitate these nanoparticles. It was added 20 mL of sterile distilled water to the 250 mL of borosilicate beaker and dissolved in 80 mL of copper sulfate [CuSO_4_] and zinc acetate [Zn(CH_3_CO_2_)_2_] as shown in Figure 1. A few modifications were made to the previous method. Stirring was carried out continuously with 20 mL of *Tribulus terrestris* aqueous extract added to the salt mixture at a temperature of 60°C (Semerdjieva and Zheljazkov 2019). The solution was then treated with 0.1 M of NaOH after 1 h. Two hours were then spent in continuous stirring; we also maintained a pH range of 12 by monitoring the pH value. It was shown in Figure 1 that the color changed from blue to darkish green, which is an optical proof that nanoparticles had formed. When copper nanoparticles are doped, the outermost layer of electrons in their *d*‐orbits overlap easily with those in the ZnO valance bond. An ethanol and acetone centrifuge were used to separate the centrifuged solution after aging overnight. Figure 1 shows the final precipitate after drying and calcining for 2 h at 400°C. As a result, greenish black fine powder was collected and stored for further processing.

### 2.4 Characterization Studies

A variety of analyses were performed on the biologically synthesized compounds, including XRD analysis, FTIR analysis, SEM analysis, UV–vis analysis, and DLS analysis. In addition to UV–vis spectrophotometer measurements, the synthesized sample was evaluated by a Perkin Elmer—Lambda 35 spectrophotometer. From 200 nm up to 800 nm is the wavelength range where absorption peaks can be measured. Synthesized nanoparticles can be analyzed using this spectroscopic method to determine their optical properties and bandgap energies. Compound chemical compositions and crystal structure can be determined by powder X‐ray diffractometer analysis. Analysis was conducted using the Bruker‐D8 in advance diffractometer. Using the PXRD spectrum, the data were collected from the angle 2θ range of 10^o^ to 90^o^. A Perkin Elmer Fourier transform infrared spectrometer was used to determine whether functional groups were present in the synthesized material. The Micrometrics—Nano plus particle size analyzer was used to determine the average particle size. An electron microscope (CAREL ZEISS‐EVO 18) was used to study the morphology of the synthesized material.

### 2.5 Antibacterial Activity

In addition to affecting human activity, bacteria also play an important role in the environment. Enterobacter, Vibrio, and Pyogenic Cocci, which are the most common bacterial pathogens causing typhoid, pneumonia, and tetanus, pose a major public health threat. A variety of natural sources produce alkaloids, a class of potentially effective natural antibiotics. Typical therapeutic strains are resistant to antibiotics, and they have a broad range of antibacterial activity (Raju et al. 2022; Ponkumar et al. 2023). In a disc diffusion experiment using Mueller‐Hinton agar, Zn‐astrakanite/CuO/ZnO nanocomposite was used to study the antibacterial properties of Gram‐negative *Escherichia coli* and Gram‐positive *Staphylococcus aureus* bacteria. After the freshly prepared media had been poured into the petri plates, new cultures of bacteria were dispersed over the plates using the spread plate technique. The petri plates were filled with standard discs of 6 mm diameter containing 20 g of nanoparticle samples. During the incubation period of 24 h, these dishes were incubated at 37°C. A millimeter‐scale measurement of the zone of inhibition was performed following incubation.

### 2.6 Dielectric Spectroscopy

By using broadband dielectric spectroscopy, the material is studied in the frequency range of 10^−6^ to 10^12^ Hz when exposed to electromagnetic radiation. The dielectric spectroscopy technique uses electrodes housing the material to determine the substance's electrical properties by observing the connection between voltage and current (*magnitude and phase*). This research was carried out using the NOVO—CONTROL −80 Broad Band Dielectric Spectrometer. An analysis based on frequency dependence was conducted for the entire research. In this case, we changed the frequency, and the electrical properties changed as a result.

## 3 Result and Discussion

### 3.1 PXRD Analysis

**FIGURE 2:**
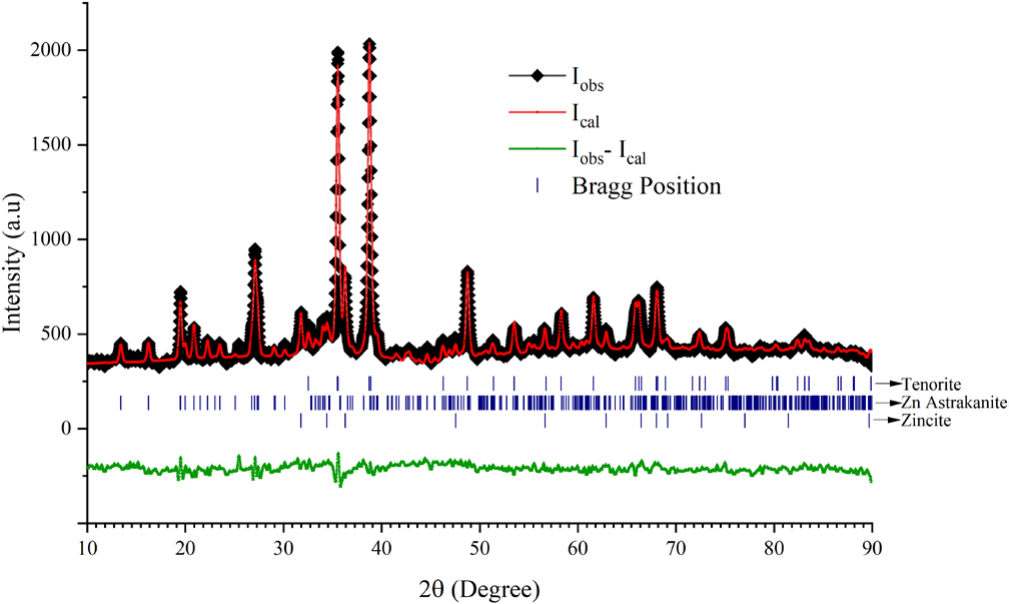
PXRD graph of biosynthesized Zn‐astrakanite/CuO/ZnO nanocomposites using *Tribulus terrestris* . Rietveld refinement revealed that the synthesized composite contains CuO, Zn‐astrakanite, and ZnO with compositions 56.26%, 31.49%, and 12.26%, respectively.

**FIGURE 3:**
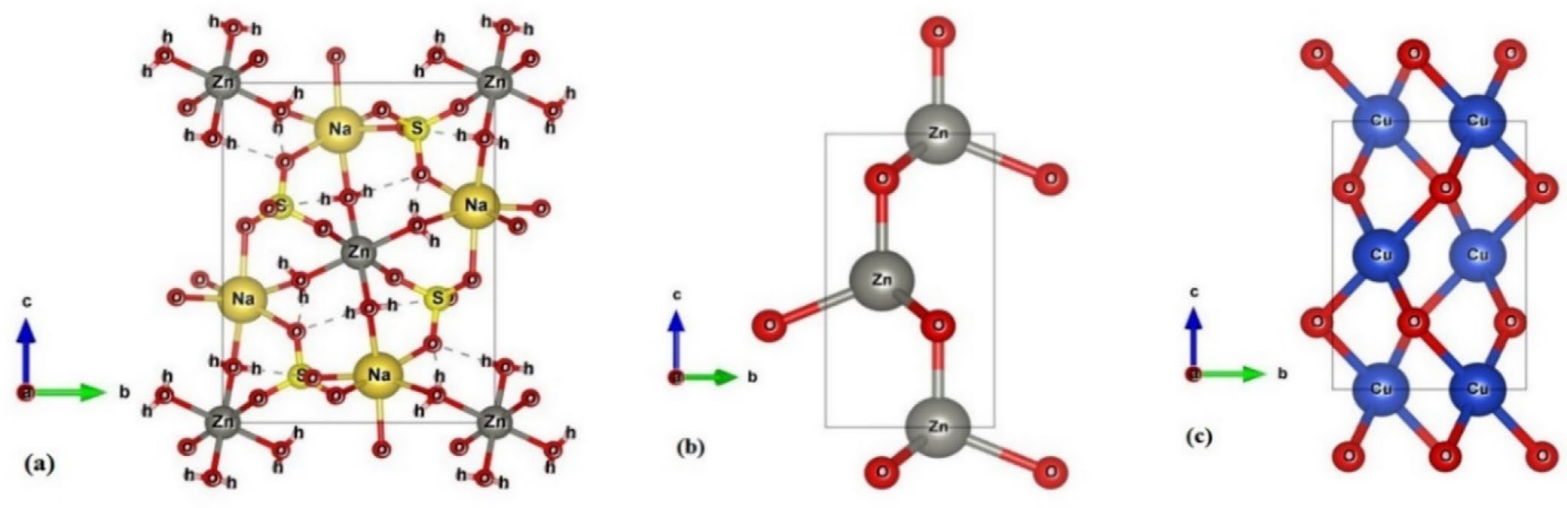
Structural images of observed nanoparticles, (a) Zn astrakanite, (b) ZnO, and (c) CuO nanostructures.

It is commonly used to determine the structure of nanocrystalline samples by using powder X‐ray diffraction (PXRD), which uses the X‐ray diffraction technique to obtain structural information. Three different nanocomposite structures were detected through the analysis of the synthesized sample. Mixed crystalline structures are obtained as a result. We present a novel method for refining disodium zinc bis(sulfate) tetrahydrate at low temperature. Zn‐astrakanite is a new crystalline form of sodium zincate Na_2_[Zn(OH)_4_] and is an upgrade of data previously reported (Murmu et al. 2011). In addition to Mg (*the original astrakanite mineral*) Co, and Ni, this compound belongs to an isostructural family of compounds. In fact Na_2_[Zn(OH)_4_] is the primary mineral astrakanite, where M = Zn (the structural properties were found in 1958 by Ruma‐Nova). Astrakanite Na_2_[Zn(OH)_4_] was examined structurally for 50 years before being structurally characterized. Herein, we demonstrate that astrakanite can be formed via biological synthesis (*Rietveld refinement entry no 96–221‐8399*). Thus, original mineral astrakanite has never been discovered by biological methods in the past. An entire family of isostructural structures has been discovered as a result of this research. In addition, 96‐900‐8878 gives us zinccite (or) ZnO structural parameters as well as tenorite (or) CuO crystalline structure. Using the results solved from the Rietveld refinement software, a PXRD graph was plotted in Figure 2. Unit cell structure of zinc astrakanite, zinccite (ZnO) and tenorite (CuO) is shown in Figure 3a–c. Phase positions are identical to those of standard phases. As shown in Tables 2 and 3, all observed structures have lattice parameters and Cartesian coordinated positions. It is a novel and rarely observed structure that Zn astrakanite has been described in the literature. As far as green synthesis is concerned, this is the most significant accomplishment. This structure has never been reported previously using a biological method of synthesis, to our knowledge.

**TABLE 2:** Cartesian coordinates position occupied by CuO, Zn astrakanite, and ZnO atomic units in the unit cell box ranges are *x* = −1.1; *y* = −1.1 and *z* = 0.1.

Atom	Cartesian coordinates Position		
	*X*	*Y*	*Z*
*CuO*			
Cu_1_	0.25000	0.25000	0.00000
O_2_	0.00000	0.41840	0.25000
*Zn astrakanite*			
Zn_1_	0.00000	0.00000	0.00000
Na_2_	0.12607	0.07173	0.36217
S_3_	0.37405	0.28842	0.13609
O_4_	0.35160	0.27120	0.26765
O_5_	0.20850	0.41630	0.07871
O_6_	0.31860	0.13129	0.07174
O_7_	0.63500	0.32955	0.13056
O_8_	−0.12470	0.03807	0.16331
O_9_	0.17530	−0.21442	0.08065
H_10_	−0.21500	−0.03200	0.17900
H_11_	−0.20700	0.12300	0.15600
H_12_	0.29900	−0.20300	0.13410
H_13_	0.21300	−0.27800	0.03200
*ZnO*			
Zn_1_	0.33333	0.66667	0.00000
O_2_	0.33333	0.66667	0.34500

**TABLE 3:** lattice parameters of CuO, Zn astrakanite, and ZnO nanocomposites.

Formula	Space group	a (Å)	α (degree)	b (Å)	β (degree)	c (Å)	γ (degree)
CuO (Tenorite)	‐C2yc	4.6884	90°	3.42108	99°	5.12656	90°
Zn‐astrakanite	‐P2ybc	5.5303	90°	8.2550	100°	11.0840	90°
ZnO (Zincite)	P6c‐2c	3.24687	90°	3.42687	90°	5.20393	120°

### 3.2 FTIR Analysis

**FIGURE 4:**
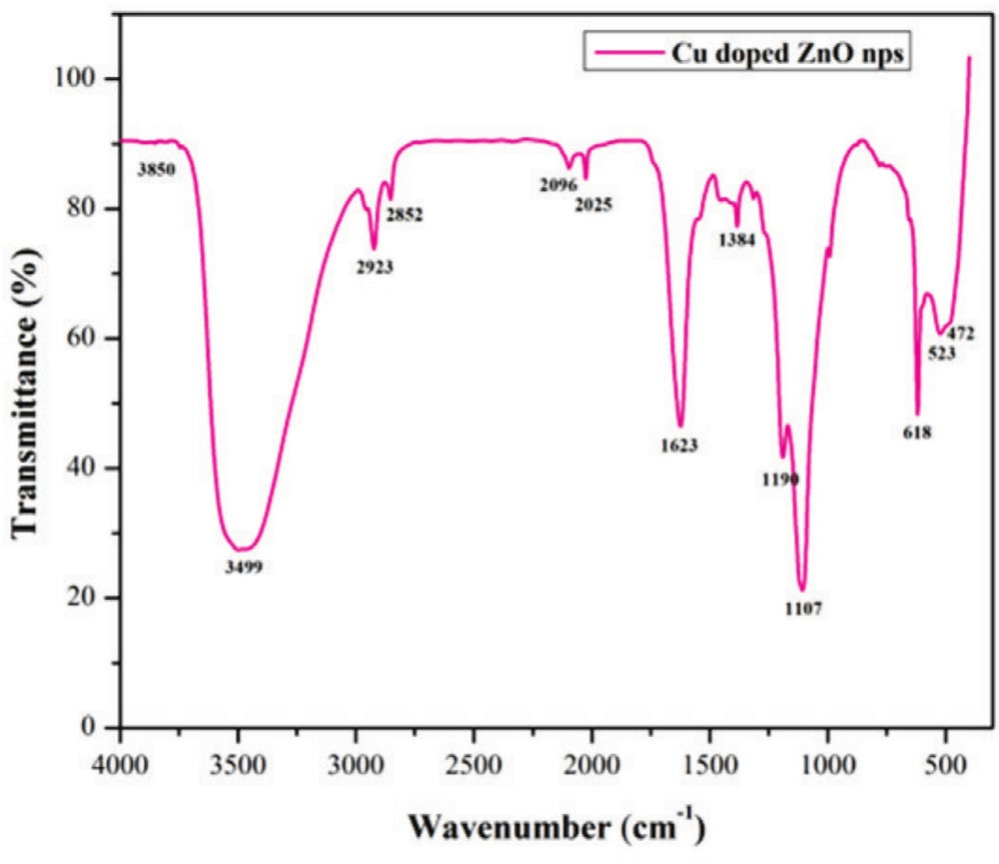
FTIR spectrum of biosynthesized Zn‐astrakanite/CuO/ZnO nanocomposite using *Tribulus terrestris*.

Functional information about nanoparticles can be determined by Fourier transform infrared (FTIR) analysis. As they are synthesized and stabilized through biomolecules in the plant, the nanoparticles are highly dependent on them. As a result of the IR results, we identified the peaks at 3499 cm^−1^, 2923 cm^−1^, 2852 cm^−1^, 2025 cm^−1^, 1623 cm^−1^, 1190 cm^−1^, 1107 cm^−1^, 523 cm^−1^, and 472 cm^−1^, which are displayed in Table 4. A strong intensity peak at 3499 cm^−1^ indicates that phenolic compounds are stretching their O‐H groups (*hydroxyl groups*). Peaks at 2923 cm^−1^ result from stretching of C‐C bonds in alkenes, and at 2852 cm^−1^ from stretching of C‐H bonds in aldehydes (*carboxylic acids*). C‐H bending vibrations of aromatics can be observed at 2025 cm^−1^, and C=O stretching of amides can be observed at 1623 cm^−1^. C‐O stretching can be seen at 1190 cm^−1^ for carbohydrates, while C‐N stretching can be seen at 1107 cm^−1^ for aliphatic amines, as shown in Figure 4. A narrow band of (O‐C=O) Cu‐O and Zn‐O lattice can be observed in the metal oxide region at 523 cm^−1^ and 472 cm^−1^, which indicates the presence of nanoparticles made up of copper and zinc oxides.

**TABLE 4:** FTIR analysis detected functional groups.

S no.	Observed peak position (cm^−1^)	Mode of vibration	Functional group	References
1	3499	O‐H stretching	Phenol and alcohol	Farag (2006)
2	2923	C=C stretching	Alkene groups	Sun et al. (2014)
3	2852	C‐H stretching	Aldehyde (carboxylic acid)	Vinayagam et al. (2022)
5	2025	C‐H bending	Aromatic compounds	Kaewta and Tanrattanakul (2008)
6	1623	C=O stretching	Amides	Ismail et al. (2014)
7	1190	C‐O stretching	Carbohydrates	Vindhya et al. (2019)
8	1107	C‐N stretching	Aliphatic amines	Getie et al. (2017)
9	523	Stretching vibrations	Cu‐O bonds	Nemade and Waghuley (2015)
10	472	Stretching	Zn‐O lattice	Al‐Hakkani (2020)

### 3.3 UV–Vis Spectroscopy and Dynamic Light Scattering Analysis

**FIGURE 5:**
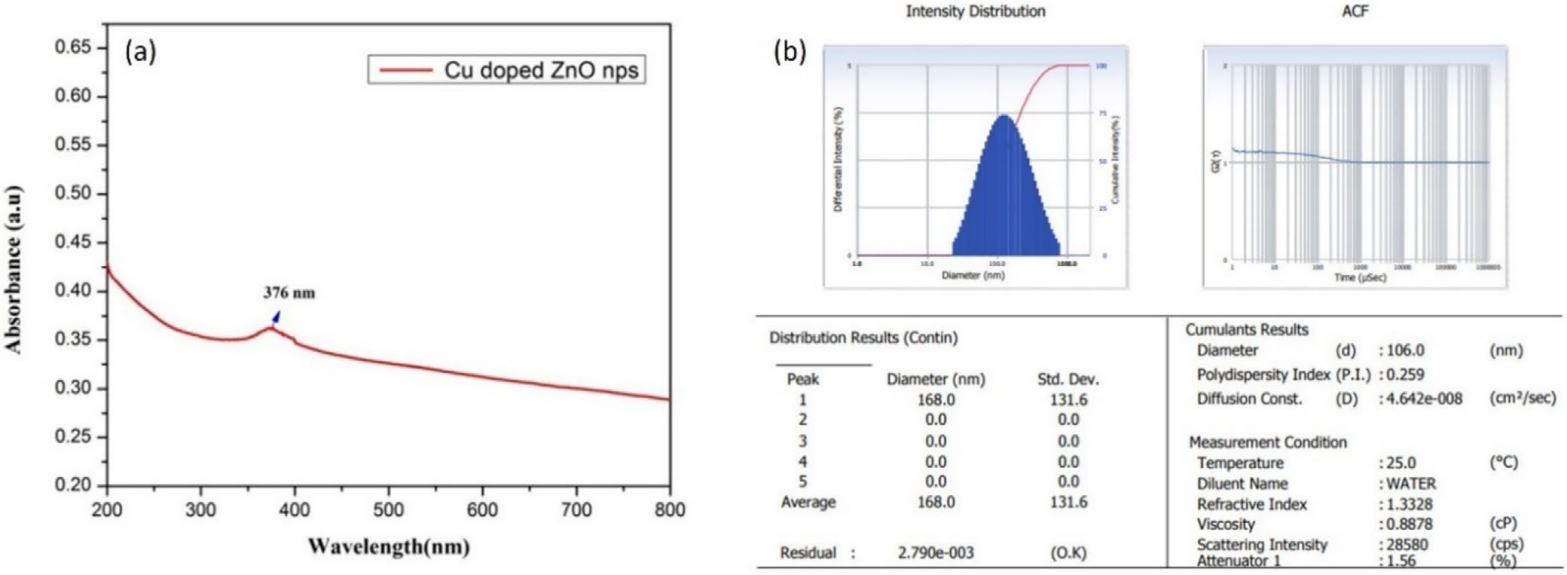
(a) UV–vis spectrum; (b) DLS of biosynthesized Zn‐astrakanite/CuO/ZnO nanocomposite nanocomposites using *Tribulus terrestris*.

In general, CuO nanoparticles in a colloidal solution absorb energy from the UV portion of electromagnetic radiation, causing an absorption peak in the range of 250 nm–350 nm in the UV–vis spectrum. In addition, the absorption area appearing between 600 nm and 800 nm is due to the transition of electrons from the lower‐nergy *d*‐orbitals to higher‐energy *d*‐orbitals in the Cu^2+^ions, which depends on the size of the CuO particles. The ZnO nanoparticles in a colloidal solution absorb energy from the incident radiation in the range of 350 nm to 390 nm. In this study, the maximum absorption peak, shown in Figure 5a, was observed at 376 nm in the UV–vis spectroscopy analysis of the prepared nanocomposite. This peak indicates the presence of ZnO nanoparticles as the major component. The color changes of the colloidal solution were used to identify the formation of copper oxide nanoparticles. The green color appearance in Figure 1 reveals the presence of CuO nanoparticles. Several reasons are expected for the appearance of green color in the colloidal solution. Due to absorbance, the transition of electrons occurs from O 2p orbitals (*valence band*) to Cu 3d orbitals (*conduction band*), resulting in a greenish appearance. It is highly expected that surface plasmon resonance (SPR) is also responsible for the green color appearance. Based on the following equation (Sarkar et al. 2020), it is possible to determine the bandgap energy of the synthesized Zn‐astrakanite/CuO/ZnO nanocomposite.
(1)

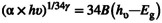




where ‘h’ indicates the Planck constant; ‘υ’ represents the photon's frequency; ‘E_g_’ indicates the bandgap energy; and ‘B’ indicates a constant. Whereas the ‘γ’ factor depends on the nature of the electron transition, and it is either 1/2 or 2 for direct and indirect transition bandgaps. Based on Equation (1), the bandgap is calculated to be ~3.30 eV. The observed peak at 376 nm in this study consistent with previous reports, where it was observed at 367 nm (Hamad et al. 2023). Due to the decrease in the particles' bandgap energy, the absorption band exhibits a slight redshift in this study (Singh et al. 2019). Dynamic light scattering analysis (DLS) is used to determine the particle size of the synthesized compound. Using the synthesized ZnO nanoparticles, the size distribution was found to be between 20 and 120 nm, as shown in Figure 5b. This sample had an average particle size distribution of 53 nm, which was calculated as the value of size distribution.

### 3.4 SEM Analysis

**FIGURE 6:**
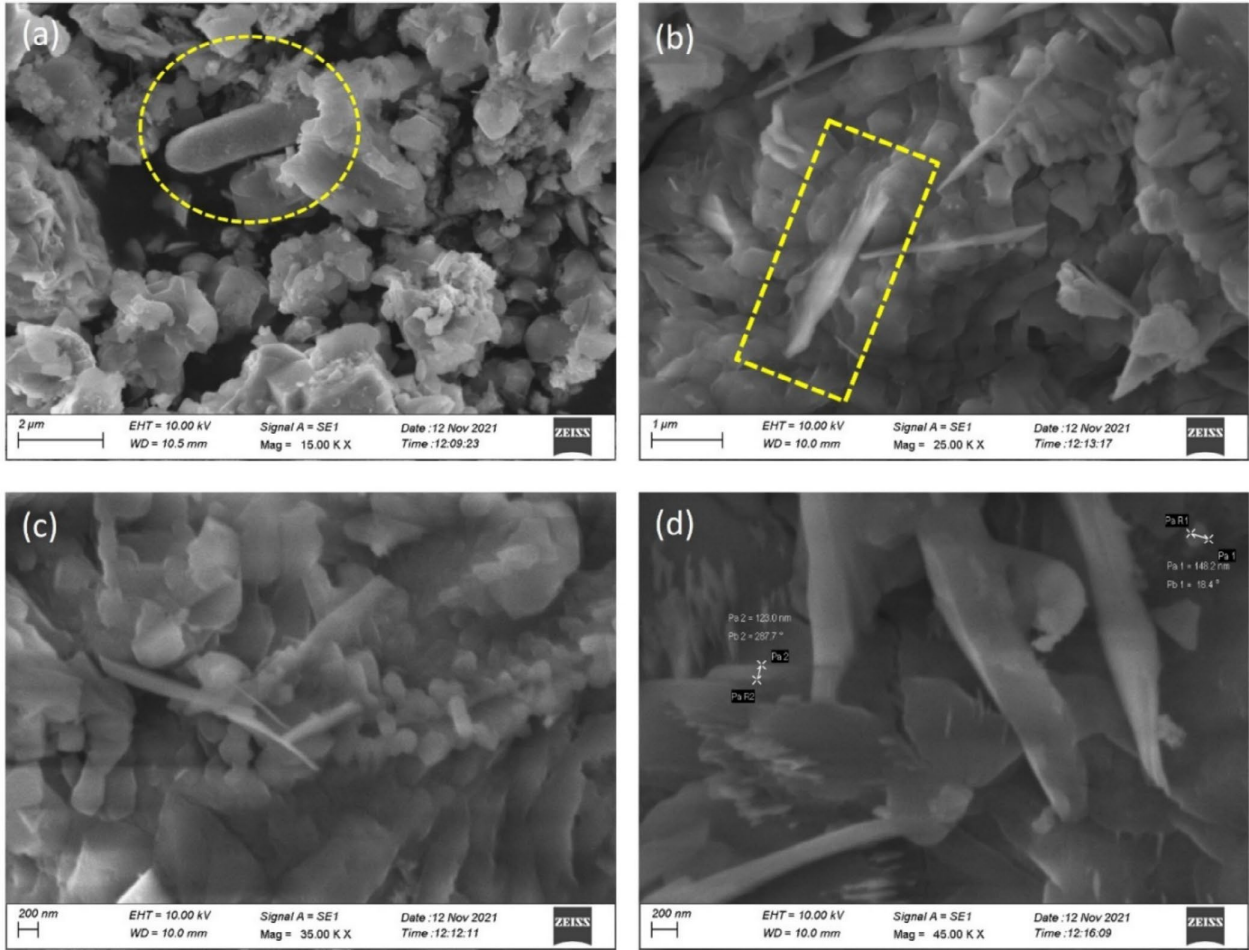
(a–d) SEM images of biosynthesized Zn‐astrakanite/CuO/ZnO nanocomposite using *Tribulus terrestris* at different magnifications.

Scanning electron microscopy (SEM) is a technique used to scan an object's surface, which provides a detailed and enlarged image. The SEM produces images through the use of a concentrated electron beam and provides details about the physical attributes and composition of the object. Atoms on the sample's surface are impacted by electron beams as they scan its surface. Sample‐specific signals are created as a result of this interaction, such as backscattered electrons, secondary electrons, and X‐rays. There is a resolution of higher due to the interactions between backscattered electrons and the sample, which are created below few microns in size. By interacting very sensitively with the surface of the sample, secondary electrons originate within a few nanometers and provide topographic details. A sample's elemental composition can be determined by X‐rays. By using a microscope with high‐resolution detectors, images can be viewed on a computer screen (Choudhary and Patri 2009). By using the scanning electron microscope, the morphological study was carried out on a Zn‐astrakanite/CuO/ZnO nanocomposite compound that was synthesized biologically. There were various magnifications used to observe the results of the Zn‐astrakanite/CuO/ZnO nanocomposite, such as 15.00, 25.00, 35.00, and 45.00 KX, as shown in Figure 6a–d. There are clusters of images, edges, and layers like nanostructures, as well as some sharp edges. As a last result, at 45 KX magnification, thin nanosheets or blade‐ike structures were observed, and the size range was calculated to be around 123 nm to 148 nm.

### 3.5 Antibacterial Activity

**FIGURE 7:**
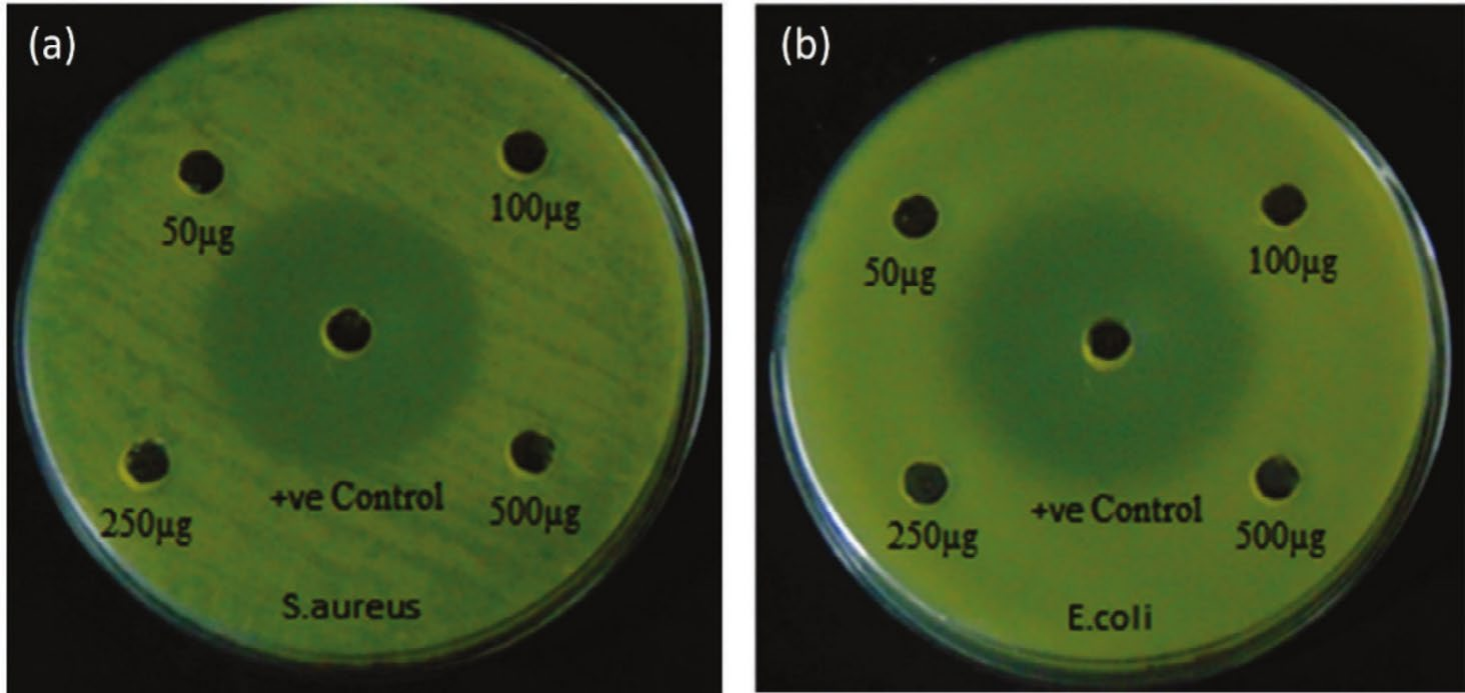
(a–b) Antibacterial activity of biosynthesized *Staphylococcus aureus* (gram‐positive) and *Escherichia coli* (gram‐negative) of Cu doped ZnO nanocomposites using *Tribulus terrestris*.

A gram‐negative and gram‐positive pathogen was used to examine the bactericidal activity of *Tribulus terrestris* capped ZnO nanoparticles. Figure 7a,b shows the resulting zone of ions resulting from *Staphylococcus aureus* and *Escherichia coli* used as gram‐positive and gram‐negative pathogens, respectively. Various concentration ranges of inhibitors are used to test the pathogen's zone of inhibition, including 50 μg, 100 μg, 250 μg, and 500 μg. The compound synthesized kills both *E. coli* and *S. aureus* bacteria at the point of sample immersion region. That region of the disc shows a transparent view because there are no bacteria there. The concentration that led to clear inhibition was above all concentrations, which suggests a good level of bacterial resistance. A significant antibacterial effect of the synthesized nanoparticles can be demonstrated by it.

### 3.6 Dielectric Analysis

**FIGURE 8:**
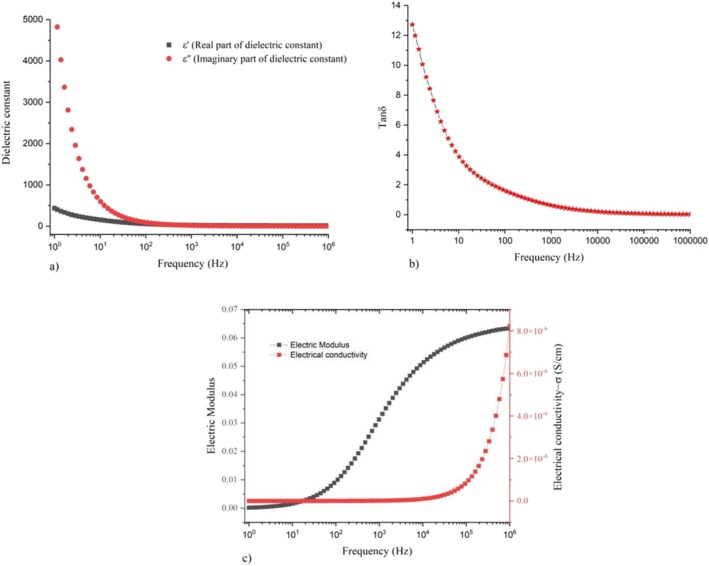
(a) Dielectric constant; (b) Dielectric loss; (c) Electrical modulus and electrical conductivity of biosynthesized Zn‐astrakanite/CuO/ZnO nanocomposite using *Tribulus terrestris*.

It is possible to obtain information about the composition, grain size, grain boundaries, transport properties, and charge storage capacity of dielectric materials by conducting dielectric studies. In addition to preparation method and chemical compounds, there are several factors that influence dielectric behavior (Ghodgaonkar et al. 1989). An electric field is applied to a substance to allow it to store electrical energy, which is called the dielectric constant. Essentially, the dielectric constant is the ratio between the permittivity of a medium and the permittivity of the surrounding space. Based on the relation given below (Acree Jr. and Yang 2017),

(2) $$k=\varepsilon_{r}=\frac{\varepsilon_{m}}{\varepsilon_{0}}$$

where ‘k’ is the dielectric constant, ε_r_ is relative permittivity, ε_m_ is a permittivity of medium and $\varepsilon_{0}$ permittivity of free space the value of dielectric constant for vacuum is 1. Generally, insulators have a dielectric constant greater than or equal to one, all other dielectric materials have the dielectric constant is greater than one that is, ε_r_ > 1. There is no unit and no dimension to the dielectric constant. During the synthesis, Cu doped ZnO particles were fabricated as pellets approximately 0.5 mm thick and kept inside between two electrodes. At room temperature, the electric field progressively increased with increasing frequency. It was necessary to monitor the conducting behavior and collect the output data. A plot of the observed data for the imaginary and real parts of the dielectric constant is shown in Figure 8a. Black color square patterns describe the real part of the dielectric constant, while red color circle patterns represent the imaginary part of the dielectric constant. On the graph, it can be seen that the real part of the dielectric constant rises with frequency at low frequencies. A decrease in dielectric constant occurs when the frequency of the electric field increases. When the electric field frequency is increased, the dielectric constant decreases because its imaginary part is very high at low frequencies. The decrease occurs because of the space‐charge polarization effect, after which it remains almost constant. The separation of mobile positively and negatively charged particles by applying an electric field causes polarization to take place, which is referred to as space charge polarization and interfacial polarization. Due to this, the prepared sample has a high dielectric constant at low frequencies. Dielectric materials conduct electrical currents and store electrical energy as a result of ac currents applied to them. Dielectric loss occurs when some electrical energy dissipates in the form of heat energy above the process. By applying the formula (Kalaiyarasi et al. 2024).

Dielectric loss and tangent factor are calculated for a material.

(3) $$\tan\delta =\frac{\varepsilon^{\prime \prime }}{\varepsilon^{\prime }}=\frac{1}{\mathrm{RC\omega}}$$

where, $\;\varepsilon^{\prime }$, $\;\varepsilon^{\prime \prime }$, R, ω and tanδ are real part of dielectric constant, imaginary part of the dielectric constant, resistance, angular frequency, and dielectric loss respectively. It is based on the frequency used and the characteristics of the dielectric material, which determines the dielectric loss. In Figure 8b, a graph of dielectric loss is shown. Our sample has an initial high dielectric loss at low frequencies and gradually decreases with increasing frequencies, as indicated by the red color stars on the graph. High frequency electric fields have a very low dielectric loss or almost constant dielectric loss. The electric modulus $M^{\prime }$ and electrical conductivity $\;\sigma^{\prime }$ must been studied and which was plotted in Figure 8c. From the graph, the black color square pattern represents electric modulus M' and red color square pattern represents electrical conductivity. It is also common for the electric modulus and electrical conductivity to be very low at low frequencies. Initially, the electric modulus was very low in the frequency region between 10^o^ Hz and 10^1^ Hz, and gradually increased from 10^1^ to 10^2^ Hz, followed by a rapid increase from 10^2^ to 10^6^ Hz with higher potential. Consequently, Cu doped ZnO nanomaterials have a higher electrical modulus with increasing frequency. When the frequency is raised above 10^5^ Hz, the material exhibits rapid electrical conductivity, indicating that it has high electrical conductivity $\sigma^{\prime }$ at higher frequencies. In addition, the electrical conductivity of the material is very low or constant up to 10^5^ Hz, but it becomes rapidly above this frequency.

## 4 Conclusion

We synthesized Cu doped ZnO nanoparticles from *Tribulus terrestris* aqueous extract and studied their properties using UV–vis, XRD, FTIR, and SEM. Biosynthesized Cu doped ZnO nanoparticles displayed a maximum peak at 376 nm, and the bandgap energy was calculated to be ~2.73 eV through UV–vis characterization. FTIR analysis revealed metal oxides and functional groups in the biosynthesized nanoparticles. Zn‐O and Cu‐O lattices were observed at 472 cm^−1^ and 523 cm^−1^, respectively. PXRD analysis revealed a new structure called Zn astrakanite, CuO (*Tenorite*), and ZnO (*Zincite*). From SEM imaging, it was revealed that the nanostructure resembled a blade. As a last step, *S. aureus and E. coli* were found to exert antibacterial activity. We observed the maximum inhibition zone at different concentrations, such as 50 μg, 100 μg, 250 μg, and 500 μg, indicating that the synthesized compound has good antibacterial properties. We conclude that the green synthesized Cu doped ZnO nanoparticles have been tested using various techniques, and results are reported. When tested under dielectric analysis, it has good dielectric properties at low frequencies and good electrical conductivity at higher frequencies.

## Author Contributions

**M. Kalaiyarasi:** conceptualization, investigation, writing – original draft, writing – review and editing, formal analysis, data curation, software, resources. **M. Mani:** conceptualization, investigation, data curation, software, methodology, visualization, project administration, formal analysis, supervision, writing – original draft, resources. **R. Harikrishnan:** project administration, writing – review and editing, investigation, validation, methodology, visualization, data curation, software, formal analysis. **N. Bharathiraja:** investigation, funding acquisition, validation, writing – review and editing, data curation. **J. Kishorkumar:** supervision, resources, data curation, writing – review and editing, software. **L. Sibali:** funding acquisition, writing – review and editing, visualization, formal analysis, software, resources, data curation. **K. Kaviyarasu:** supervision, funding acquisition, writing – review and editing, resources, data curation, formal analysis, software, investigation, conceptualization.

## Conflicts of Interest

The authors declare no conflicts of interest.

## Data Availability

The data that support the findings of this study are available from the corresponding author upon reasonable request.
